# Abusive Supervision and Its Impact on Knowledge Hiding Behavior Among Sales Force

**DOI:** 10.3389/fpsyg.2021.800778

**Published:** 2021-12-30

**Authors:** Rana Faizan Gul, Liu Dunnan, Khalid Jamil, Fazal Hussain Awan, Basharat Ali, Ayaz Qaiser, Qi Aobin

**Affiliations:** ^1^School of Economics and Management, North China Electric Power University, Beijing, China; ^2^Beijing Key Laboratory of New Energy and Low-Carbon Development, North China Electric Power University, Beijing, China; ^3^Department of Sociology, Government College University Faisalabad, Faisalabad, Pakistan; ^4^Department of Business Administration, University of Sialkot, Sialkot, Pakistan

**Keywords:** abusive supervision, knowledge hiding, psychological contract breach, evasive hiding, playing dumb, rationalized hiding

## Abstract

The purpose of this study is to test the relationship between abusive supervision and employee’s knowledge hiding behavior (evasive hiding, playing dumb, rationalized hiding) among sales force of insurance companies in Pakistan. The paper also strives to theoretically discuss and then seek empirical evidence to the mediational paths of psychological contract breach that explain the focal relationship between abusive supervision and knowledge hiding. To test the proposed hypotheses, the study draws cross-sectional data from sales force of insurance companies working in Pakistan. Data were collected through structured questionnaire and using convenient sampling technique. The final sample of 340 valid and complete responses analyzed using structured equation modeling (partial least square) approach. Results showed that abusive supervision is positively related to employee’s knowledge hiding behaviors. Also, mediating variable psychological contract breach partially mediates the abusive supervision-knowledge hiding behavior linkage. Current study has tested the positive relationship between abusive supervision and knowledge hiding behaviors unlike most of the previous investigations that have focused on knowledge sharing behavior. The study also empirically investigated the mediational route of psychological contract breach, that explains the blame attributed by the beleaguered employee that led to covert retaliatory behavior, such as knowledge hiding. This paper contributes to knowledge hiding literature which is an important part of knowledge management from the perspective of abusive supervision based on both reactance theory and SET theory.

## Introduction

Every organization needs knowledge since it provides sustainable competitiveness in the current complex, ambiguous, uncertain, and volatile world. Employees ought to acquire and share knowledge amongst themselves (Han et al., 2021). A diverse and organization with a greater capacity emphasis on the adoption of the various strategic mix, such as the knowledge management (KM). KM helps companies respond to transitions and boost activities’ viability and competitive advantage. In designing and implementing innovative services and goods, KMP has been viewed as a critical component to handle the business method in the modern business environment. This is why companies continue to implement new and successful knowledge management methods to achieve sustainability goals (Kumar Jha and Varkkey, 2018). Therefore, organizations are expected to invest in creating a comprehensive knowledge management system besides ensuring there is a conducive environment that promotes goodwill and trust to have seamless sharing of knowledge. According to several studies, transferring knowledge from one employee to another enhances organizational performance and effectiveness (Singh, 2019).

Despite efforts made by organizations to ensure there is the proper transfer of knowledge among employees, there is still some reluctance among employees to share knowledge (Singh, 2019). Most organizations have gone the extra mile to put strategies that will ensure a smooth transfer of knowledge amongst employees. The measures include introducing systems that reward employees, strengthening and enhancing interpersonal relationships around the workplace, and creating a culture that enhances sharing of knowledge (Xiao and Cooke, 2019). Nevertheless, some employees have chosen not to share some of their critical knowledge with others. Despite the numerous studies conducted to demystify the importance of knowledge sharing, many employees are still rigid and fond of hoarding knowledge (Di Vaio et al., 2021).

Knowledge hiding is influenced by organizational factors, knowledge content, and individual factors. Based on personal factors, the contributors of knowledge hiding include perceived knowledge value, psychological possession of knowledge, and commitment to knowledge commitment (Nguyen et al., 2022). According to Connelly et al. (2012) factors such as corporate rules, reward systems and leadership may have an impact on how information is hidden. Sharing information at work is influenced by interpersonal relationships and the way one is treated by their supervisors. The present research is mostly quiet on how dysfunctional leadership influences an employee’s choice to withhold information from others. On factors related to the knowledge, you find knowledge complexity and task relatedness. Other organizational factors that lead to knowledge hiding include politics, goal orientation, policies, knowledge management systems, culture, and leadership styles (Miminoshvili and Černe, 2021). As a result, you will find individuals who do not want to share knowledge avoiding activities that involve sharing of knowledge. Also, according to Khoreva and Wechtler (2020), abusive behaviors displayed by supervisors facilitate the habit of hiding knowledge. Although, such a topic is still lacking in terms of research since not many people have investigated how abusive behaviors by supervisors lead to knowledge hoarding (Anser et al., 2021).

Supervisors’ abusive behaviors include intimidating, ridiculing, criticizing, and undermining employees’ perceptions regarding the psychological contract (Khalid et al., 2018). The abusive behavior is always evident through hostile verbal and non-verbal behaviors but without physical contact. Once the psychological contract of employees has been breached, they opt to withhold knowledge (Jahanzeb et al., 2019). However, studies on the connection between knowledge hoarding and abusive supervision are still limited. But the consequences of abusive supervision can be dealt with through individual characteristics (Ghani et al., 2020).

Most of the studies have focused on the association between knowledge sharing behaviors and supervisor abuse that is different from knowledge hoarding behavior regarding the employee’s motivation and intention (Feng and Wang, 2019). Our study is focused on both knowledge management and abusive supervision. The research aims to unravel the relationship between knowledge hiding behaviors and abusive supervision. In this research, hoarding of knowledge is regarded as covert retaliation used by the employee in the event of abusive supervision. According to Agarwal et al. (2021a,b)), when an aggravated person has less positional power than the supervisor or organization, where supervisory abuse is coming from, the individual will tend to use subtle retaliatory and overt strategies instead of direct retaliation overt. The study goes to the extent of investigating the knowledge hiding behaviors among employees, which often go undetected. However, they have adverse effects on the proper functioning and performance of a firm such as innovativeness, sustainability, profitability, and productivity (Xiao and Cooke, 2019).

The study also seeks to investigate to whom the blame goes in the light of abusive supervision that causes knowledge hiding behavior. According to Khoreva and Wechtler (2020), the employee subjected to abusive supervision will either resolve to blame the supervisor who dispensed the abuse or the organization for failing to lay down measures that protect the interests of employees. To demystify the indirect impact of knowledge hoarding behavior and abusive supervision, this research considers literature that clarifies if the employee will blame the organization in the instance of supervisory abuse.

There is a relevant literature review in the subsequent paragraphs, followed by a hypothesis of the relationships between the focal constructs. After which comes the research methodology, then analyses, and then discussion of results. The last section involves the implications, limitations, and future research recommendations for management.

## Hypotheses and Development and Literature Review

### Components of Knowledge Hiding

Connelly et al. (2012) expressed that the knowledge hiding is, “intentionally attempting to hide or conceal task information, ideas, or know-how that has been sought by another person.” When employees believe their immediate supervisor or managers are authentic and transformational, they are more willing to share critical resources with other members of the organization, whereas when they believe their immediate supervisor or managers are toxic and destructive, they are more reluctant to share their knowledge and demonstrate knowledge hiding behaviors. The three forms of knowledge hiding include evasive hiding that entails misleading pledge to offer a full answer in the future or offering wrong information; rationalized hiding that entails blaming others or justifying your failure to provide the needed knowledge; and playing dumbs whereby the person hiding knowledge ignores the request to share knowledge (Weng et al., 2020). In knowledge hiding, unlike other negative knowledge behaviors, there is always a clear intent for an individual to decline sharing the requested information (Butt and Ahmad, 2021). On the other side, knowledge withholding entails an individual providing less information than the one required. Thus, it can be done intentionally since the person might not be sure that they are withholding important knowledge (Gul et al., 2021; Moh’d et al., 2021). For knowledge hoarding, the knowledge gathered might not have been requested. So, this study focuses on demystifying Intra organizational hiding of knowledge with great emphasis on the individual hiding of knowledge requested to supplement the knowledge associated with the study (Zhang and Min, 2021).

### Social Exchange Theory

We find theoretical support for our argument in SET (Blau, 1968), which predicts knowledge concealing in the presence of abusive supervision. Individual activities motivated by a desired outcome are referred to be SET. For instance, an employee who goes above and beyond the call of duty expects the business to recognize and reward him or her appropriately (Gouldner, 1960). The social trade is governed by the reciprocity standard, which establishes the acceptable behavior of the participating parties. Positive and negative reciprocity norms are possible. Positive reciprocity entails a favorable reaction to favorable treatment, while negative reciprocity entails a tendency to react adversely to adverse treatment (Cropanzano and Mitchell, 2005). Thus, when an individual employee believes that he or she is being treated unfavorably, the individual will act unfavorably in return as a kind of reciprocity.

Social exchange theory also suggests that supervisory abuse leads to knowledge hiding. Usually, an individual’s actions will be determined by a particular return being sought. According to Cook et al. (2013), an employee who goes the extra mile expects reward or recognition from the organization. So, it is evident that social exchange depends on a reciprocity norm which reveals the proper way of behaving (Mohsin et al., 2021; Sukumaran and Lanke, 2021). This norm can be not only positive but also negative (Bogilović et al., 2017). Positive reciprocity entails a positive response in the event of positive treatment, while on the other side negative reciprocity involves a negative response when there is negative treatment (Lanke, 2018). As a result, when an employee undergoes unfavorable treatment, he or she might respond negatively, which can be in the form of hiding knowledge (Cropanzano et al., 2002).

### Abusive Supervision and Knowledge Hiding

According to past studies, supervisory abuse emanates from employees’ undesirable behavioral and attitudinal outcomes like increased deviant workplace behavior, emotional exhaustion, job burnout, reduction in the organization’s citizenship behavior, and low job performance (Arain et al., 2020). The abusive behaviors displayed by supervisors include hostile behaviors, belittlement, public ridicule, rudeness, and mocking. Besides, other studies also tested various individual (psychological entitlement and professional commitment) and organizational factors (leadership styles, policies, and organizational cultures) to be antecedents with knowledge hiding (Abdillah et al., 2020).

Thornton et al. (2009) revealed that knowledge hiding is the deliberate effort by one person to hide information demanded by another individual. Knowledge hiding behaviors were classified into three categories: rationalized hiding, playing dumb, and evasive hiding. In rationalized hiding, a person attempts to justify himself or herself to the information seeker or to blame a third party for failing to provide the desired knowledge. Similarly, when a person plays dumb, he or she portrays ignorance of the information sought by the knowledge seeker. Evasive hiding is described as the concealer supplying false facts or promising to disclose information in the future in order to deceive the knowledge seeker with no such genuine motives.

One of the undesirable behavioral outcomes happens to be knowledge hiding, which tends to be ignored in most literature about abusive supervision. Employee knowledge hiding behavior is one of the many outcomes of supervisory abuse. Many studies about abusive supervision regard it as a cause of various negative workplace results (Lin et al., 2020; Awan et al., 2021). However, several practitioners and scholars assume that knowledge sharing, and knowledge hiding are opposite outcomes from a similar continuum. But according to Rezwan and Takahashi (2021), these two are different constructs with different underlying mechanisms, antecedents, and motivations. The literature on the way and reasons as to why employees hide knowledge is lacking, unlike in studies demystifying the reasons why and how people share knowledge (Oubrich et al., 2021; Sarfraz et al., 2021).

Due to stronger decisional power and high power in organizations, employee engagement, and important duties in achieving organizational goals, leaders tend to display supervisory abuse such as attributing undesirable results, taking credit on behalf of employees, intimidating, ridiculing, and yelling at employees (Zhao and Jiang, 2021). As a result, supervisory abuse causes destructive leadership, whereby the leader displays both verbal and non-verbal hostile behaviors but without physical contact.

According to Lanke (2018), there are ways through which the negative outcomes of supervisory abuse can be established, especially through establishing the relationship between knowledge-associated behaviors and abusive supervision. Some ways include having a supportive organization that prevents the negative effects of supervisory abuse that affects knowledge sharing (Butt, 2020; Jamil et al., 2021). Islamic work ethics also weaken the direct negative impact of supervisory abuse that causes knowledge hiding (Khalid et al., 2018). According to Di Vaio et al. (2021), organizational justice also remedies supervisory abuse that prevents knowledge sharing because of emotional exhaustion. Group trust also weakens the negative impact brought about by supervisory abuse that prevents sharing of knowledge due to psychological capital (Nguyen et al., 2022). Self-reliance and psychological contract fulfillment can also reduce supervisory abuse on sharing knowledge by the leader to member exchange (Xiao and Cooke, 2019). But studies on how organizational factors like motivation climate that is likely to cause hiding of knowledge either directly or indirectly are still lacking in extant literature (Han et al., 2021).

Supervisory abuse is a negative leadership trait that causes various deleterious and harmful work outcomes to an organization and an individual (Fischer et al., 2021). Employees only tend to share critical information with others when they find the managers or supervisors to be transformational or authentic (Islam et al., 2021). But when the supervisor happens to be destructive and toxic, employees will be reluctant to share knowledge hence displaying knowledge hiding actions (Khalid et al., 2018). Studies conducted reveal that when a supervisor is abusive, there will be some form of retaliation from the employees searching for fairness. The employees will retaliate as revenge for the abusive supervision displayed by the supervisor or manager (Ambrose and Ganegoda, 2020). But when the retaliation is direct and overt, it will not do any good for the employee due to some restraining factors like differences in positional power and the organizational hierarchy (Naseem et al., 2020; Teng et al., 2021). In return, the employee will resolve to use covert retaliation, which champions creating fairness without getting punished or identified (Low et al., 2021). Hence, we proposed following hypotheses:

H1a: Abusive Supervision has significant relationship with evasive hiding

H1b: Abusive Supervision has significant relationship with playing dumb

H1c: Abusive Supervision has significant relationship with rationalized hiding

### Mediating Role of Psychological Contract Breach

A psychological contract breach (PCB) is a cognitive perception showing that an employee is yet to receive everything promised to them by the organization either formally or informally. The PCB occurs when an organization or their representatives, for instance, managers, do not live up to the employee’s expectations. Usually, a psychological contract refers to an individuals’ beliefs shared by an organization regarding the conditions of exchange agreement spelled out between an organization and an employee (Vogelgesang et al., 2021). It refers to an employee’s belief about the obligations explicitly or implicitly, or formally or informally made by the organization they are working for. Whenever the employee feels like the organization is not honoring its promises or commitments, they will likely feel betrayed and consider a psychological contract breach (Zacher and Rudolph, 2021). The employees who are subjected to abusive supervision are the ones likely to experience psychological contract breaches. The abusive supervisors always degrade, belittle, mock, and display related hostile behaviors to employees (Vogelgesang et al., 2021).

Employees expect proper and fair treatment at the workplace; therefore, when the employee feels like they are being abused or mistreated by the organization’s representative, such as a manager, he or she will consider it unjust treatment and a grave breach of faith (Kaya and Karatepe, 2020). To the employee, if the manager or supervisor who is a representative of the organization acts abusive, then he or she holds the thought that the entire organization has indeed breached the contract on respectful, just, and fair treatment. In this regard, the organization will be considered a serious culprit, whereas the manager or supervisor is a representative who exercises abusive supervision on behalf of the organization (Karatepe et al., 2021). Besides, the employee will also lay the blame on other employees for not supporting or helping him or her in such an ordeal. The aggrieved employee might even consider other employees to be equally guilty of supervisory abuse in the case that they are not supporting him or her (Jayaweera et al., 2021). As a result, such an employee resolves to hide knowledge from others. Ampofo (2021) stated, coworker support is likely to compensate for the outcomes of abusive supervision. According to DiFonzo et al. (2020), there is a relationship between knowledge hiding and abusive supervision. The procedural justice theory states that a person who receives constant abuse and humiliation from a supervisor will certainly believe that an organization is not doing enough to create and enforce measures to protect the aggrieved employee or punish rogue supervisors (Agarwal et al., 2021b). As a result, an employee who is exposed to consistent supervisory abuse will display knowledge hiding behavior, which will not do any good in stopping mistreatment but instead will be like supporting the perpetrator of the supervisory abuse (Arunachalam, 2021).

Psychological contract breach emanates from employee performance, civic virtue behaviors, employee commitment, employee intentions to remain in the organization, citizenship behaviors, job satisfaction, and trust in the management (Stanway et al., 2020). However, PCB is also likely to be supported by revenge cognitions, higher absenteeism, employee cynicism, job burnout, neglecting job duties, and deviant behaviors and the workplace (Kraak et al., 2020). Hence, we proposed following hypotheses:

H2a: Psychological contract breach mediates the relationship of abusive supervision and evasive hiding

H2b: Psychological contract breach mediates the relationship of abusive supervision and playing dumb

**FIGURE 1:**
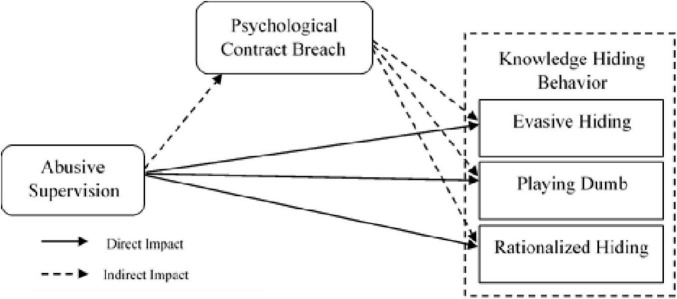
Conceptual framework.

H2c: Psychological contract breach mediates the relationship of abusive supervision and rationalized hiding (Figure 1 show all the relationships)

### Research Methodology

We gathered data from the sales employees of insurance companies working in Pakistan. Data were collected from employees working in all four provincial capitals (i.e., Lahore, Karachi, Quetta, Peshawar) as well as national capital Islamabad. The motive behind the selection of sales employees of insurance companies is that these employees have to face strict targets and most of the time they face short of targets which leads to face abusive behavior from their supervisors. A convenient sampling approach was employed (Kothari, 2004). The overall sample size of this research was 340 sales employees of insurance companies. A total of 460 questionnaires were distributed, and 355 responses were received. There were 340 appropriate replies for the final analysis, with a response rate of 73.9%. Valid questionnaires are selected following the survey data cleaning procedure, which involves finding and eliminating responses from respondents who either do not meet our target requirements or did not react cautiously to the questionnaire survey, such as respondents only address part of our survey; respondents provide ambiguous answers or/and select the same answer option repetitively, and respondents provide incomprehensible suggestions for open-ended questions.

### Measures

The study used items established from prior research to confirm the reliability and validity of the measures. All items are evaluated through five-point Likert-type scales where “1” (strongly disagree), “3” (neutral), and “5” (strongly agree). To analyze the three dimensions of knowledge hiding, we used twelve items adopted from prior studies. Evasive Hiding is determined by four items adopted from the studies of Connelly et al. (2012) and a sample item is “I agree to help other team members but intentionally not provide valuable information.” Playing Dumb is evaluated by four items adapted from the work of Thornton et al. (2009) and a sample item is “When I ask some important information from my team member, he/she pretended that I did not know the information related to your work.” Rationalized Hiding is measured by four items and adopted from the studies of Demirkasimoglu (2016) and a sample item is “When my team member asked me the required information, I explained that the information is confidential and only available to people on a particular project.” To get response about independent variable we used eleven items adopted from the prior study of Tepper (2000), and the sample item is, “My boss is rude to me.” Psychological contract breach was used as mediating variable. For the measurement of psychological contract breach, we used six items adopted from the study of Robinson and Wolfe Morrison (2000) and the sample item is, “I have not received everything promised to me in exchange for my contributions.”

## Results

Smart PLS 3.2.9 software package was utilized for this research (Sarstedt et al., 2014). PLS-SEM analysis consists of two stages: the first is an evaluation of the measurement model, and the second is an evaluation of the structural model (Henseler et al., 2009). The measurement model requirement ensures that only constructs with adequate indicator loading, convergent validity, composite reliability (CR), and discriminant validity will be included in the structural model. The structural model assessment aims to determine path coefficients and evaluate their significance using the boot-strapping method. Concerning mediation assessment, Preacher and Hayes (2008) technique was pursued in the present study. It is the more exact method for determining mediating effects and is more compatible with the PLS-SEM method (Hayes, 2009). The majority of recent research studies in the field of knowledge management have used the PLS-SEM software to assess data (Shujahat et al., 2019; Sahibzada et al., 2020).

### Common Method Bias

Common method Bias variance (CMV) is a critical concern when conducting survey research. When data was gathered from a single source, this problem arose (Podsakoff et al., 2012). A single-factor test was used to determine the existence of CMV among variables, as Harman (1976). proposed. Harman single-factor analysis is a *post hoc* technique for determining if a single factor accounts for employees’ silence in a data collection (Tehseen et al., 2017). The “Harman’s single-factor test” was conducted in this study using SPSS 25. The results obtained using the principal axis factoring and extraction approach revealed 31 unique factors. The first unrotated component accounted for just 34.910 percent of the variation in the data set, less than the 40% stated by Hair Joe et al. (2016) (see Appendix 1). Additionally, we ran a complete collinearity evaluation test in Smart PLS as Kock (2015), and many social science experts have proposed (Zafar et al., 2020). All VIF values are less than 5, indicating that common method bias is not a problem (Kock, 2015).

### Measurement Model Assessment

**FIGURE 2:**
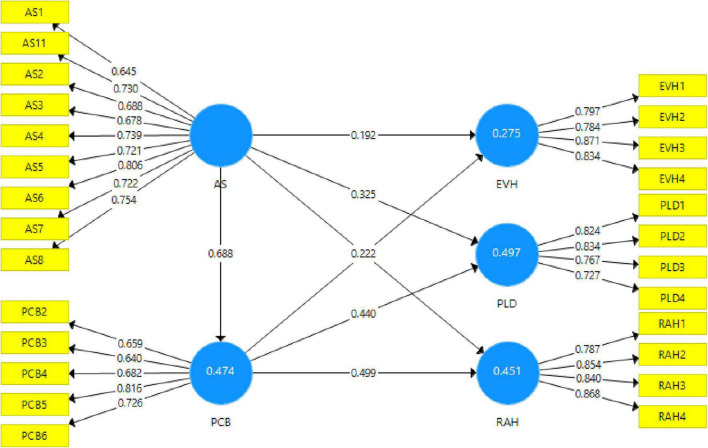
Measurement model.

The first phase evaluated the measuring model to validate the constructs’ reliability and validity (Hair et al., 2014). We ran consistent PLS algorithm to validate the reflective constructs. Individual items’ reliabilities are assessed through factor loadings of the items on the corresponding constructs. Only those Items containing factor loading equal to or greater than 0.6; have been considered significant and retained in the model and the Cronbach’s alpha values of all constructs are greater than the suggested threshold of 0.7, which are acceptable (Sarstedt et al., 2014). Additionally, for further strengthens the assessment of the reliability of the construct. The composite reliability of the constructs is also assessed because it is commonly admitted that composite reliability is a more effective tool to measure the reliability than Cronbach’s alpha (Werts et al., 2007). The composite reliability values of all the constructs are also greater than 0.7, which further toughens the reliability of all the variables (see Table 1 and Figure 2 for detailed values).

**TABLE 1:** Reliability and validity measures.

Constructs	Items	Loadings	VIF	*T*-value	Cα	CR	AVE
Abusive supervision					0.865	0.865	0.517
	AS4	0.656	1.522	11.377			
	AS5	0.729	1.824	15.151			
	AS6	0.693	2.629	16.507			
	AS7	0.650	2.095	13.557			
	AS8	0.800	1.960	18.506			
	AS11	0.773	1.713	16.611			
Psychological contract breach					0.806	0.802	0.504
	PCB3	0.709	2.231	13.303			
	PCB4	0.647	2.555	11.706			
	PCB5	0.743	1.929	15.526			
	PCB6	0.736	1.201	16.611			
Evasive hiding					0.839	0.837	0.563
	EVH1	0.672	2.248	9.148			
	EVH2	0.749	2.511	12.527			
	EVH3	0.752	1.737	11.455			
	EVH4	0.821	1.694	13.977			
Playing Dumb					0.846	0.845	0.579
	PLD1	0.843	2.060	17.610			
	PLD2	0.804	1.764	17.771			
	PLD3	0.688	1.872	11.928			
	PLD4	0.696	1.961	14.009			
Rationalized hiding					0.858	0.859	0.606
	RAH1	0.656	1.707	10.024			
	RAH2	0.785	2.123	17.401			
	RAH3	0.821	2.085	19.050			
	RAH4	0.839	2.311	20.097			

The Fornell–Larcker criteria and heterotrait–monotrait (HTMT) ratios are used to validate the current study’s discriminant validity (Hair Joe et al., 2016). According to the Fornell and Larcker (1981) criteria, if each column’s upper side initial value is greatest after calculating the square root of the AVE of each variable, discriminant validity has been established (Fornell and Larcker, 1981; Hair Joe et al., 2016). According toTable 2, discriminant validity has been demonstrated using the Fornell–Larcker criteria since the top value of variable correlation in each column is the greatest. According to the HTMT ratios criteria, HTMT ratios should be less than 0.85; nevertheless, values up to 0.90 are acceptable (Hair Joe et al., 2016). As shown in Table 2, all HTMT ratios are less than the recommended threshold, indicating that discriminant validity has been established for the current research model. The values that lie in off-diagonal are smaller than the average variance’s square root (highlighted on the diagonal), supporting the scales’ satisfactory discriminant validity. Consequently, the outcome affirmed that the Fornell and Larcker (1981) model is met.

**TABLE 2:** Discriminant validity.

Fornell-Larcker criterion						Heterotrait-Monotrait ratio (HTMT)					
	AS	EVH	PCB	PLD	RAH		AS	EVH	PCB	PLD	RAH
AS	0.719					AS					
EVH	0.542	0.751				EVH	0.541				
PCB	0.663	0.555	0.710			PCB	0.654	0.545			
PLD	0.703	0.463	0.664	0.761		PLD	0.694	0.461	0.661		
RAH	0.646	0.587	0.656	0.742	0.779	RAH	0.647	0.587	0.649	0.742	

*AS, Abusive Supervision; PCB, Psychological Contract Breach; EVH, Evasive Hiding; PLD, Playing Dumb; RAH, Rationalized Hiding.*

Moreover, this research examined the variance inflation factor (VIF) values to confirm the model’s collinearity problems. Experts believe that if the inner VIF values are less than 5, there are no collinearity problems in the data (Hair et al., 2014). According to the findings of this research, the inner VIF values of constructs are less the suggested cut off value. Thus, it demonstrates no collinearity issue in the current study’s data and validates the model’s robustness. The *R*2 and *Q*2 values of Psychological Contract Breach 0.439 (*Q*2 = 0.184), Evasive Hiding 0.362 (*Q*2 = 0.177), Playing Dumb 0.564 (*Q*2 = 0.2855), and Rationalized Hiding 0.510 (*Q*2 = 0.272), which support the model’s in-sample predictive power (Sarstedt et al., 2014); and The results of blindfolding with an omission distance of seven provide *Q*2 values considerably above zero (Table 3), confirming the model’s predictive relevance in terms of out-of-sample prediction (Hair et al., 2014).

**TABLE 3:** Coefficient of determination and predictive relevance.

Endogenous constructs	*R* ^2^	*Q* ^2^
Psychological contract breach	0.439	0.184
Evasive hiding	0.362	0.177
Playing dumb	0.564	0.285
Rationalized hiding	0.510	0.272

### Assessment of Structural Model

In the second phase of PLS SEM, structural model was assessed. The consistent PLS bootstrap resampling technique with 5,000 resamples (Hair Joe et al., 2016) was utilized to establish the significance of direct and mediating relationships. Tables 4, 5 lists the test results of hypotheses intended for direct and indirect associations.

**TABLE 4:** Direct effects.

Hypotheses	Statistical paths	Beta	2.5%	97.5%	*P* values	*T* statistics	Conclusion
H1a	AS → EVH	0.312	0.129	0.500	0.001	0.312	Supported
H1b	AS → PLD	0.468	0.303	0.643	0.000	0.468	Supported
H1c	AS → RAH	0.376	0.210	0.549	0.000	0.376	Supported

*AS, Abusive Supervision; EVH, Evasive Hiding; PLD, Playing Dumb; RAH, Rationalized Hiding.*

**TABLE 5:** Direct, indirect, and total effects.

	Direct effects		Indirect effects		Total effects		
Indirect path	*B*	*t*	β	*t*	*B*	*T*	Conclusion
AS -> PCB -> EVH	0.312	3.279	0.231	3.103	0.542	8.613	Partial Mediation
*BCI LL*	0.129		0.090		0.422		
*BCI UL*	0.500		0.379		0.664		
AS -> PCB -> PLD	0.468	5.320	0.235	3.967	0.703	15.115	Partial Mediation
*BCI LL*	0.303		0.120		0.613		
*BCI UL*	0.643		0.353		0.794		
AS -> PCB -> RAH	0.376	4.374	0.270	4.270	0.646	13.540	Partial Mediation
*BCI LL*	0.210		0.148		0.554		
*BCI UL*	0.549		0.395		0.738		

*AS, Abusive Supervision; PCB, Psychological Contract Breach; EVH, Evasive Hiding; PLD, Playing Dumb; RAH, Rationalized Hiding; BCI LL, Bootstrapped Confidence Interval Lower level; BCI UL, Bootstrapped Confidence Interval Upper level.*

**FIGURE 3:**
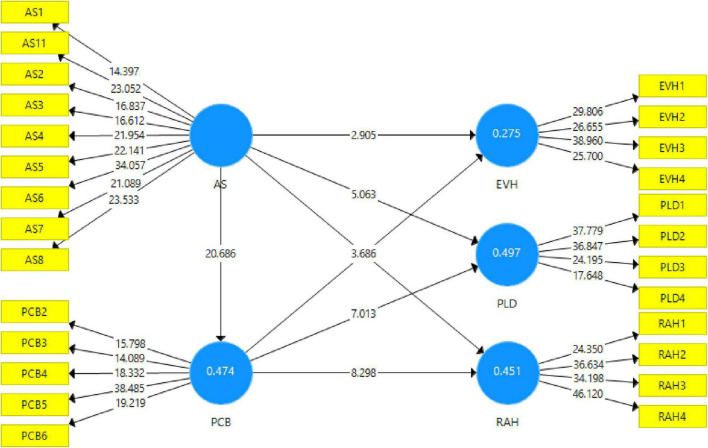
Structural model.

As shown in Table 4 and Figure 3, hypotheses H1a, H1b, and H1c related to Abusive supervision’s positive effect on three aspects of knowledge hiding behavior, namely Evasive Hiding, Playing Dumb, and Rationalized Hiding. The findings indicate that Abusive supervision has significant associations with all the three aspects of knowledge hiding behavior. Specifically, Abusive supervision’s influence on playing dumb (β = 0.468; *p* < 0.001) is more significant than its effect on Evasive Hiding (β = 0.312; *p* = 0.004) and Rationalized Hiding (β = 0.376; *p* < 0.001). Hence, H1a, H1b, and H1c are accepted.

Next to test the three mediating hypotheses, we performed the mediation analysis in Smart-PLS using the Hayes and Preacher (2010) bias-corrected bootstrapping approach at a 95% confidence interval.

H2a, H2b, and H2c the mediating role of Psychological contract breach in the relationship between abusive supervision and three dimensions of knowledge hiding (Evasive hiding, playing dumb, and Rationalized Hiding) was proposed. For H2a, Results reveal that Psychological contract breach significantly mediate the relationship between abusive supervision and Evasive hiding (β = 0.231; *t* = 3.130), playing dumb (β = 0.235; *p* = 3.967) and Rationalized Hiding (β = 0.270; *t* = 4.270) which supports H2a, H2b, and H2c. Moreover, it can be seen that psychological contract breach partly mediates the association between abusive supervision and three dimensions of knowledge hiding namely, evasive hiding, playing dumb, and Rationalized Hiding, as seen in Table 5.

## Discussion

Our study aims to establish the relationship between abusive supervision on employees and knowledge hiding behaviors such as rationalized hiding, playing dumb and evasive hiding. The study also seeks to explore psychological contract breach to determine who the employee is likely to blame in the event of mistreatment and its impact on knowledge hiding. Despite the fact, there is an association between abusive supervision and knowledge hiding, the extent of literature is still lacking since the only available empirical evidence can be found in Khalid et al. (2018) study. For that reason, our research is determined to expound on the relationship between abusive supervision and knowledge hiding. Our findings are supported by the social exchange theory, which states that the employee tends to retaliate in the event of abusive supervision. Still, upon the realization that the perpetrator holds high positional power, the employee is likely to use a covert and safer retaliatory strategy such as knowledge hiding. The finding of this research also gets support from reactance theory, whereby an employee who experiences supervisory abuse tends to have limited personal control while at the workplace, this finding is parallel with the prior study of Ghani et al. (2020). Such employees will opt to take part in activities that will provide them with some sense of control to overcome the frustrations resulting from abusive supervision (Feng and Wang, 2019). In that regard, the employee will decide to hide or withhold information that might be valuable to the organization just because of having been subjected to supervisory abuse.

The study also explains the mediating role of psychological contract breach between abusive supervision and knowledge hiding behaviors like rationalized hiding, playing dumb and evasive hiding. According to the findings, psychological contract breach affects abusive supervision and knowledge hiding behavior. In a psychological contract breach, an employee regards the supervisor or manager as a representative of the organization; therefore, the organization ought also to be held equally responsible for the abuse in the instance of supervisory abuse. The employee is likely to complain that the organization has not laid down measures to prevent the occurrence of supervisory abuse, neither is it showing any support for the employee. As a result, the employee resolves to withhold crucial information that is valuable to the organization. Such an outcome confirms fairness heuristic theory, which explains how psychological contract breach affects abusive supervision and knowledge hiding this finding is in line with the previous study of Agarwal et al. (2021a).

## Theoretical Implications

This study has several theoretical implications. Negative knowledge behaviors, i.e., information concealment, are our first addition to the literature in the field of knowledge management. The majority of prior research have concentrated on knowledge sharing, which is a positive knowledge-related activity, and have ignored knowledge hiding. Although many firms have implemented various knowledge management systems, they will not be as effective if we don’t understand why employees choose to keep information from their peers a secret. Therefore, investigations on knowledge concealing are critical to the advancement of KM theory and practice. To better comprehend the negative intra-organizational knowledge-related behavior, this research attempts to investigate it and adds to the knowledge management literature.

The study also provides crucial theoretical implications through investigating the association between covert retaliation from employees and abusive supervision. This study aims to explain why the victim of abusive supervision decides to hide knowledge when subjected to abusive supervision in their workplace. Unfortunately, noticing the knowledge hiding traits can be a hard nut to crack for the supervisors. Therefore, it can be a challenge to issue additional punishment. Besides, due to exercising discretion, the employee might hide knowledge, and such an act will not be taken seriously by the manager or supervisor. This study also seeks to determine who holds the blame in the event of supervisory abuse at the workplace. Normally it is the manager, supervisor, coworkers, and organization. As a result, the employee seems to have gotten their revenge following the abusive supervision she was subjected to by hiding knowledge.

## Practical Implications

According to the findings, there are various theoretical and managerial implications. Supervisory abuse affects many organizations and has negative effects, such as negatively impacting the profitability of an organization (Miminoshvili and Černe, 2021). The most affected organizations are those that are knowledge intensive. Such organizations are likely to fall prey to the employees’ toxic behavior of intentionally hiding knowledge due to abusive supervision and other interpersonal animosities. Some industries at risk of suffering the negative effects of abusive supervision include the banking sector, which explicitly depends on intense knowledge to make decisions by considering real-time information or data. Therefore, having the wrong information or dealing with employees who withhold crucial information results in dire consequences. Organizations affected by abusive supervision and knowledge hiding are bound to become less competitive since they are deprived of the crucial knowledge to give them an edge over their competitors.

The impacts of knowledge hiding can seriously hurt the business in terms of innovation and creativity, whereby there is too much hoarding of information and secrecy. According to Khoreva and Wechtler (2020), it is quite challenging to eradicate knowledge hiding from an organization. But the organization can put in place measures to ensure such an interpersonal nuisance is not affecting the organization. Some of the measures are through ensuring that employees are treated fairly in the workplace so that they have no reason to be tempted into hiding critical knowledge. The organization can also opt to train and sensitize managers or supervisors to avoid mistreating, discriminating or belittling employees. Lastly, the organization can also offer support and counseling services to employees who are facing abusive supervision.

## Limitations and Recommendations

Despite the study contributing immensely to demystifying the direct and indirect impact of supervisory abuse that triggers knowledge hiding traits among employees, it also has some limitations. First limitation of study is a small sample size, and cross-sectional data were used, thus making it hard to establish a relationship between the main focal constructs. Even though this study considered precautionary measures during gathering and analyzing data, still future studies are bound to integrate objective and multi-source data in a bid to expand on this relationship. It will also help if future studies determine the role played by coworkers to establish why the aggrieved employees decide to intentionally hide knowledge from other employees in the event of abusive supervision at the workplace. Another limitation is that this study collected data from services sector employees, it would be interesting if future studies will focus on other sectors such as manufacturing sector. Future studies should also explore organizational and individual factors like team dynamics, job interdependence, reward expectation, and psychological ownership. Researchers should also look at the knowledge being hidden by the employee whenever there is supervisory abuse at work. For instance, in case the knowledge happens to be discretionary or an intrinsic, then the employee will deem it best to hide it, unlike when it is extrinsic and explicit in nature.

## Conclusion

Most previous studies have not focused on knowledge hiding instead of knowledge sharing. These two focal constructs have different motivations and antecedents, so they cannot be two ends in a similar continuum. So, this research belongs to the few empirical investigations seeking to test for the positive relationship between knowledge hiding behaviors and abusive supervision. The study also sought to investigate how psychological breach contract affects abusive supervision and knowledge hiding. Psychological contract breach explains the blame that the employee lays on not only the employee but also the organization in the event of abusive supervision, thus proving the dysfunctional nature depicted in knowledge hiding.

## Data Availability Statement

The original contributions presented in the study are included in the article/supplementary material, further inquiries can be directed to the corresponding author.

## Author Contributions

All authors listed have made a substantial, direct, and intellectual contribution to the work, and approved it for publication.

## Conflict of Interest

The authors declare that the research was conducted in the absence of any commercial or financial relationships that could be construed as a potential conflict of interest.

## Publisher’s Note

All claims expressed in this article are solely those of the authors and do not necessarily represent those of their affiliated organizations, or those of the publisher, the editors and the reviewers. Any product that may be evaluated in this article, or claim that may be made by its manufacturer, is not guaranteed or endorsed by the publisher.

## Appendix

**APPENDIX 1:** Harman’s single-factor test.

Factor	Total variance explained					
	Initial eigenvalues			Extraction sums of squared loadings		
	Total	% of Variance	Cumulative%	Total	% of Variance	Cumulative%
1	10.751	37.072	37.072	10.124	34.910	34.910
2	2.192	7.558	44.630			
3	1.695	5.846	50.475			
4	1.412	4.869	55.345			
5	1.157	3.991	59.336			
6	1.059	3.653	62.988			
7	0.962	3.316	66.304			
8	0.853	2.943	69.247			
9	0.810	2.794	72.041			
10	0.797	2.749	74.790			
11	0.695	2.395	77.186			
12	0.624	2.150	79.336			
13	0.559	1.926	81.262			
14	0.528	1.821	83.083			
15	0.508	1.752	84.834			
16	0.436	1.505	86.339			
17	0.424	1.461	87.801			
18	0.410	1.414	89.214			
19	0.390	1.345	90.559			
20	0.371	1.280	91.839			
21	0.353	1.218	93.057			
22	0.339	1.168	94.225			
23	0.323	1.114	95.340			
24	0.257	0.885	96.225			
25	0.244	0.841	97.066			
26	0.234	0.806	97.872			
27	0.232	0.799	98.671			
28	0.209	0.720	99.391			
29	0.177	0.609	100.000			

*Extraction method: Principal axis factoring.*
